# Ketamine as a Bridge Therapy: Reducing Acute Suicidality in Hospital Settings

**DOI:** 10.3390/healthcare14050634

**Published:** 2026-03-03

**Authors:** Paul E. Lie, Titus Y. Lie, Madeleine Nguyen, Donald Y. C. Lie

**Affiliations:** 1 School of Medicine, Texas Tech University Health Sciences Center (TTUHSC), Lubbock, TX 79430, USA; paul.lie@ttuhsc.edu; 2 Julia Jones Matthews TTUHSC School of Population and Public Health, Texas Tech University Health Sciences Center, Abilene, TX 79601, USA; 3 Graduate School of Biomedical Sciences, Texas Tech University (TTU), Lubbock, TX 79409, USA; 4 Jerry H. Hodge TTUHSC School of Pharmacy, Texas Tech University Health Sciences Center, Amarillo, TX 79106, USA; titus.lie@ttuhsc.edu; 5 Talkington School for Young Women Leaders, Lubbock, TX 79416, USA; 6 Department of Electrical and Computer Engineering, Edward E. Whitacre Jr. College of Engineering, Texas Tech University (TTU) and TTUHSC, Lubbock, TX 79409, USA

**Keywords:** bridge therapy, ketamine, major depressive disorder, novel therapeutics, patient safety, pharmacology, psychopharmacology, SSRIs, suicide risk

## Abstract

This narrative literature review explores the clinical use of Ketamine as part of an untested hypothetical model framework for bridge therapy for acute suicidality. Long-term suicide rates continue to increase in the United States and in many other countries, creating a pressing public health challenge with a variety of treatment considerations. Existing standard-of-care SSRI therapeutics have a delay between administration and symptom relief at 2–6 weeks, leaving a so-called danger zone of about 1–3 months of risk for suicidal follow-through behaviors. Ketamine, a potent NMDA (N-methyl-D-aspartate) receptor antagonist, has recently seen widespread interest in both regulatory and clinical settings for increasing neuroplasticity and alleviating depressive symptoms. Ketamine’s mechanism-of-action through mTORC1 is much faster than SSRI’s downstream transcriptional regulation, leading to quicker relief of suicidal symptoms and the removal of the danger zone lag period. The current literature suggests that a controlled, supervised subanesthetic dose of Ketamine in a clinical setting has low risks of addiction or abuse, distinguishing therapeutic uses of Ketamine from recreational uses. While the biological efficacy of Ketamine is established, this conceptual review focuses on a possible initial hypothetical framework of a “Bridge Protocol.” We searched PubMed, Google Scholar, The Cochrane Library, and PsycINFO (January 2000–December 2025) to synthesize evidence regarding SSRI latency, acute Ketamine protocols, and post-discharge safety. We conclude that while promising, the proposed Ketamine Bridge Therapy requires rigorous longitudinal validation and sustained clinical studies before it can be safely used and experience widespread adoption.

## 1. Introduction

**Figure 1:**
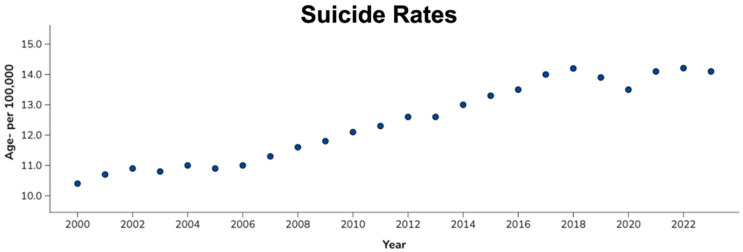
Suicide rate levels between 2000 and 2023 in the US. Note a peak in 2018, decreasing slightly through 2020, then returning to peak rates at 2021–2023. 2024 and 2025 data remain provisional and are still in processing phases [1].

Suicidality presents a pressing challenge for healthcare professionals and institutions due to increasing Major Depressive Disorder (MDD) and Post-Traumatic Stress Disorder (PTSD) as public health burdens, especially within developed nations. The last two decades have seen a consistent rise in suicide rates, rising by more than 37% between 2000 and 2025 [1], as indicated in Figure 1. The initial mandated action for a patient presenting in triage with suicidal ideation and plausible risk of harm-to-self is psychiatric hospitalization, which places institutional strain on hospitals in already underserved trauma care and stroke care areas. Large urban hospitals with significant resources may be able to tolerate some suicidal psychiatric inpatient hospitalization, but the cost of these cases may be substantial. Moreover, the current Standard of Care (SoC) of Selective Serotonin Reuptake Inhibitors (SSRIs) initiation with psychotherapy has notable limitations.

### Public Health Impact and Economic Burden of Suicidality

Suicide has substantial economic effects on the global economy and public health infrastructure. According to recent CDC data, the total combined cost of suicide and non-fatal self-injurious behavior in the US alone is over $500 billion annually [2]. This large amount includes medical costs, lost productivity/wages, and costs of quality-of-life reductions. The danger zone window between hospital discharge and SSRI therapeutic efficacy offers heightened risk during this period. Patients who are discharged from the hospitals frequently are re-admitted for follow-up suicide attempts, which constitute a burden on the public health system. Long intensive care unit (ICU) stays, emergency room visits, short-term drug administrations, and follow-up appointments can be incredibly expensive, both for the hospital and the patient. We hypothesize that a Ketamine bridge therapy could reduce the healthcare costs associated with high-risk readmissions, although rigorous public health and socioeconomic determinants of health studies are required to validate or confirm this potential benefit. However, rising suicide rates among Americans aged 10–24 severely underscore the need for new interventions that demonstrate efficacy more rapidly than the traditional 2–6-week timeline of SSRIs, such as Fluoxetine and Sertraline. In 2004, the FDA (Food and Drug Administration) issued a “black box” warning on SSRIs for youth, which led to a decrease in prescriptions of SSRIs and was temporally associated with a rise in suicide rates among young adults [3,4]. This hesitation to treat due to the side effects and latency of SSRIs may lead to adverse clinical outcomes, which we propose that Ketamine may play a role in alleviating. While the antidepressant efficacy of Ketamine is well-documented in functional studies [5,6], this review focuses specifically on its possible use as part of a larger “Bridge Protocol”. We propose this protocol for consideration to hypothetically bridge the 2–6-week therapeutic latency of SSRIs during the traditionally highest-risk post-discharge window. The main objective of this conceptual review is to reference past and ongoing clinical evidence to explore the feasibility of this Bridge Protocol to attenuate or mitigate suicide risk.

## 2. Materials and Methods

This study was designed as a conceptual review of the pertinent adjacent literature. We performed a comprehensive search of the PubMed (National Center for Biotechnology Information, Bethesda, MD, USA; https://pubmed.ncbi.nlm.nih.gov/), PsycINFO (American Psychological Association, Washington, DC, USA; https://www.apa.org/pubs/databases/psycinfo (accessed on 3 January 2026)), EBSCO (EBSCO Information Services, Ipswich, MA, USA; https://www.ebsco.com), Google Scholar (Google LLC, Mountain View, CA, USA; https://scholar.google.com), and Cochrane Library (Cochrane, London, UK; https://www.cochranelibrary.com) databases for articles that were published explicitly between January 2000 and December 2025.” We utilized Boolean operators to combine the following primary search terms: (“Ketamine”, OR “Esketamine”) AND (“Acute Depression” OR “Suicidality” OR “Hospitalization”) AND (“Bridge Therapy”, OR “SSRI Latency”). This initial database search produced 502 articles. After accounting for duplicates between databases and ensuring that papers were aligned with our relevant clinical acute psychiatric contexts, a total of 39 full-text peer-reviewed articles were included in this conceptual review. For Inclusion Criteria, we prioritized Randomized Controlled Trials (RCTs), meta-analyses, and systematic reviews published in English that specifically addressed (1) the pharmacological latency of SSRIs, (2) the rapid-acting mechanisms of Ketamine, or (3) included/measured suicide risk rates after hospital discharge. For Exclusion Criteria, we explicitly excluded single-case studies, non-peer-reviewed commentaries, non-scientific institutional websites, opinion pieces, and studies that focused on chronic or indefinite-maintenance Ketamine administration without any documented instance of acute hospitalization. This is a conceptual review, and a formal PRISMA risk-of-bias assessment was not performed.

### 2.1. Current SoC

A therapeutic latency generally exists between the administration of SSRIs and the relief of suicidal ideation and risk of self-harm. This latency is thought to be due to the initial mechanism of SSRIs, which inhibits serotonin reuptake in the synaptic cleft by serotonin transporter (SERT) but does not rapidly release more vesicular serotonin from the presynaptic neurons of the raphe nuclei. Patients may initially feel their mood paradoxically decrease in the first week, due to the increase in serotonin in the synaptic cleft, which triggers autoinhibitory receptors 5-HT1A on the presynaptic cell body, reducing serotonergic action potential firing [7,8]. Firing regulation may become normalized after a longer time, in which synaptic 5-HT ligands trigger Gs-PCR, which, in turn, increases adenylyl cyclase, cAMP secondary messengers, and subsequent intracellular PKA, translocating into the nucleus to phosphorylate the downstream transcription factor CREB. CREB binds to the promoter region of the Brain-Derived Neurotrophic Factor (BDNF) gene and increases transcription of BDNF protein. While the mechanism behind SSRI antidepressant activity is not yet fully elucidated, it is thought that BDNF plays a significant role in increasing synaptic plasticity and inducing the remodeling of actin filaments in the dendritic post-synaptic spines [7]. While these synaptic spine mechanisms appear to be directly implicated, the mechanisms of antidepressant action are multifactorial and warrant further research. The BDNF hypothesis suggests that increased levels of the neurotrophin may be associated with improved emotional well-being, while simultaneously increasing plasticity to adapt toward stress resilience and reversing stress-related cortical atrophy. While the exact component mechanisms are not yet fully understood, patients on SSRI maintenance often report a sustained improvement in mood and quality of life that slowly builds over the pharmacological timeline. Clinical expectations typically predict SSRI therapeutic efficacy and depressive symptom relief at an effective interval of between 2 and 6 weeks.

### 2.2. Danger Zone

**Figure 2:**
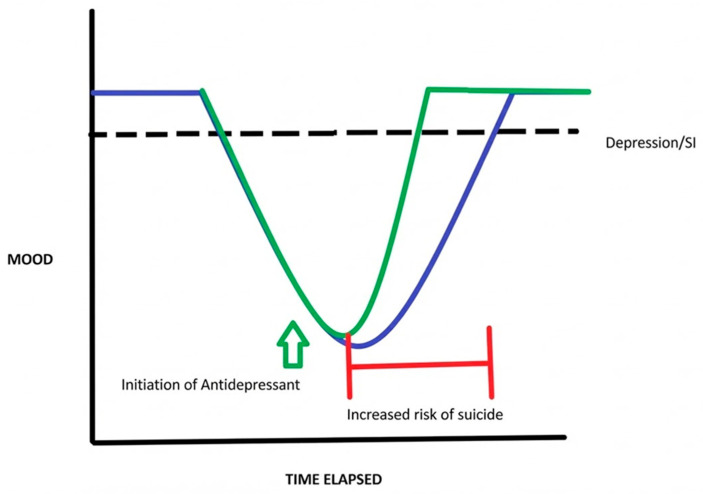
Schematic for mood fluctuation and danger zone after hospital discharge.

**Figure 3:**
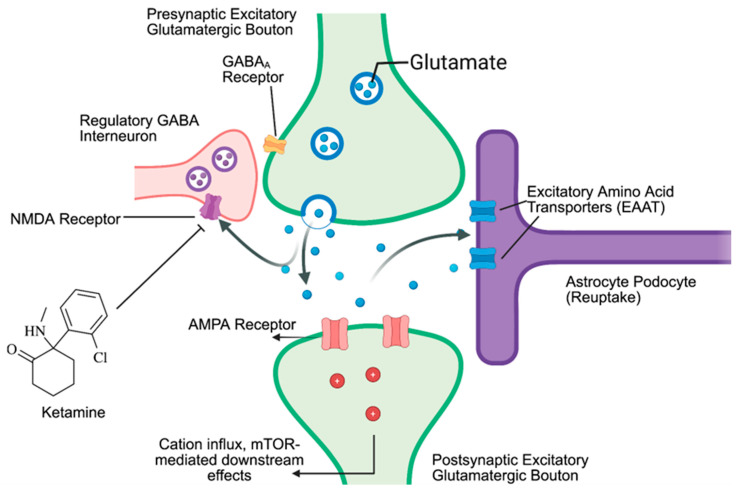
Mechanism of Ketamine on the neuronal level. Antagonism of NMDA receptors on GABAergic regulatory interneurons induces changes in excitatory glutamatergic transmission and E/I balance.

A paradoxical “danger zone” has been noted in the literature, in which patients are frequently discharged before SSRIs have taken full effect [9]. This 1–3 month post-discharge window is the period in which the patient presents the greatest risk for suicidal behavior or self-injurious behavior (SIB), as illustrated in Figure 2. A hypothetical “bridging” therapy may conceivably help to address this danger zone as a step-down therapy before SSRIs mediate their gradual transcriptional downstream effects. Ketamine, by comparison, has an observed rapid mechanism of action, shortening the timeframe for immediate depressive symptom relief to within hours. It is hypothesized that this sustained and fast effect may be mediated by bypassing complex, long-term transcriptional outcomes [10]. Ketamine’s pharmacological role as a non-competitive antagonist of the NMDA receptor has been established. In the proposed hypothetical framework, Ketamine’s NMDA blockade reduces presynaptic glutamate release and stimulates postsynaptic AMPA receptors. The resulting mTORC1 upregulation then might build analogous dendritic spines using mRNA already present within the cytoplasm, which may explain the delay in relief from weeks to hours when compared to SSRIs, though this mechanism still requires further validation [10,11]. Dendritic spine formation has been histologically observed about 2 h after Ketamine administration, which could align with patients’ reported perceptual improvements in mood, cognition, and synaptic plasticity [11]. A secondary mechanistic hypothesis has also emerged in recent years, in which Ketamine may also act on NMDA Receptors on regulatory GABAergic Interneurons, leading to increased glutamatergic signaling and synergistic indirect activation of AMPA receptors, as suggested in Figure 3. A study at the New York Psychiatric Institute found that subanesthetic Ketamine appears to increase neurocognitive functioning [12] in individuals when compared to midazolam controls. Patients who were given IV Ketamine performed significantly better (*p* < 0.05) on the neurocognitive battery, through an improvement in the choice reaction time (CRT) and interference processing on a Stroop computer test [13]. Another study by the same author concluded that the Scale for Suicidal Ideation (SSI) score was significantly lower in Ketamine patients when compared to the benzodiazepine control group, while the proportion of responders was higher at 55% vs. 30%, respectively [14]. Other systematic reviews have also reported tenuous optimism in similar anti-suicidal effects of Ketamine [15,16,17].

### 2.3. Comparative Efficacy: IV Ketamine vs. Intranasal Esketamine

A key distinction for clinical Ketamine use in our proposed framework is in the choice of agent and administration method: intravenous (IV) racemic R/S Ketamine and intranasal (IN) esketamine. The US FDA has approved Esketamine (S-enantiomer of Ketamine) under the drug name Spravato for use in Treatment-Resistant Depression (TRD) [18]. Some evidence suggests that the R-enantiomer (R-Ketamine) may have unique antidepressant properties that are beyond traditional NMDA receptor blockade, possibly through the understudied metabolite 26,6R-hydroxynorketamine [19]. However, R-Ketamine is not currently approved by the FDA as a treatment for any psychiatric conditions. IV Ketamine is racemic, comprising both R and S enantiomer forms of the compound. Some clinical data suggests that IV racemic Ketamine might potentially offer a more rapid and significant anti-suicidal effect when compared to intranasally administered Esketamine, possibly due in part to the increased bioavailability of IV administration by avoiding first-pass metabolism [20]. It was also reported that IV Ketamine appeared to have a quicker onset and a higher response rate for reducing suicidal ideation when compared to intranasal Esketamine [21,22]. While these claims require additional validation and future robust clinical studies, preliminary evidence suggests that the IV option of administration may possibly be the better choice for the proposed clinical bridge therapy framework. A potential pitfall of improved bioavailability in IV Ketamine administration, however, is both in care and cost: the procedure is inherently more invasive and may require significant hospital resources, such as a dedicated phlebotomist or nursing team to consistently monitor the patient’s IV line. Conversely, the nasal spray Esketamine’s administration format has been reported to be easier to implement for both patients and providers in outpatient maintenance settings to reduce the cost of care and staffing needs, potentially increasing access to care in medically underserved areas, despite experiencing a hypothetically reduced bioavailability [23]. Future public health socioeconomic status (SES) studies could elucidate which method is more appropriate for a given healthcare setting and client.

### 2.4. Precision Medicine: Selecting Patients

It is currently hypothesized that Ketamine mediates its observed rapid relief of depressive symptoms through the described mechanism of mTORC1-mediated dendritic spine remodeling and synaptic plasticity, but response rates are an area of concern in the population strata of patients at risk of suicide [24,25,26]. Some preliminary data suggests that a significant proportion (30%) of patients with severe Treatment-Resistant Depression (TRD) may have lower response rates to IV racemic Ketamine administrations, though the mechanism underlying this etiology is not currently known [27]. Developing a rapid biomarker predictive screening panel might help hospitals and public health officials to better determine eligibility for limited resources, manage important expectations of patients, and help with difficult healthcare professional decisions [27]. Additionally, there is early evidence that suggests Ketamine may be most effective in a subset of patients with endogenous neuroinflammation and inflammatory cytokine levels [28]. Other preliminary evidence appears to suggest that patients with higher C-Reactive Protein (CRP) and Interleukin-6 (IL-6), two classical molecules modulating inflammation, often show a more significant and robust antidepressant effect from Ketamine administration when compared to Fluoxetine or other SSRIs [28]. This observation might align with the inflammation hypothesis for Ketamine: that Ketamine possibly mediates a part of its antidepressant activity through the reduction of neuroinflammation and localized synaptic inflammation. BMI (Body Mass Index) may also be a useful clinical predictor. A meta-analysis of clinical trial data determined that patients clustered into two unique phenotypes based on BMI scores below and above 30 [27]. Those with the higher BMI (above 30) appeared to have higher response rates to IV-administered racemic Ketamine, which the authors hypothesized could be due to the redistribution of the nonpolar molecule in peripheral adipose tissue sinks. Additionally, as is similar to other pharmacological interventions, emerging evidence suggests that different single-nucleotide polymorphisms (SNPs) of specific genes appear to result in different projected outcomes with the treatment. Specifically, the substitution of Valine at position 66 with the amino acid Methionine significantly changes the BDNF (Brain-Derived Neurotrophic Factor) gene, which may play a role in modulating neuroplasticity in both SSRI and Ketamine outcomes within our wider hypothetical framework. Other SES predictive factors may also be implicated in early efficacy studies. One study observed that a history of family alcohol use may paradoxically be correlated to a more robust antidepressant response to Ketamine [29]. Future clinical research could conceivably investigate whether integrating these biomarkers into admission protocols could possibly optimize patient selection for bridge therapy. Analyzing these various SES dimensions and population-group-level stratifications may allow future clinicians to tailor treatments to their unique community and patient settings. Future studies may elucidate the utility of using public health measures and biomarkers in patient screening for the proposed conceptual Ketamine bridge framework.

### 2.5. A Note on Adolescents

The initial FDA “black box” warning on SSRIs was associated with a considerable spike in youth cohort suicide rates within the United States. Suicide continues to be the leading cause of death for individuals between the ages of 10 and 24. Ketamine, therefore, retains a possibly useful role in alleviating the suicide rates within this young age group. The results of one randomized controlled trial (RCT) [10] appeared to indicate that a single IV Ketamine racemic infusion significantly (*p* < 0.05) reduced suicidal ideation in adolescents with treatment-resistant depression when compared to benzodiazepine controls. However, certain complications may require further investigation due to the complex effect of sedatives on the emerging young adult brain. Future research could conduct robust safety trials to assess whether the use of NMDA antagonists, such as Ketamine, may affect developmental psychology and early neurogenesis [30]. The aforementioned RCT also produced evidence that the dissociative effects of subanesthetic doses of Ketamine were not subjectively stressful and had no lasting negative effects on the young patients [10]. Despite this data, the overall safety profile of Ketamine on the emerging brain is complex and may require vigorous testing before any recommendations can be made. In a testament to the multifactorial, lesser-known pharmacological effects of adolescence, another study found that the anti-depressive effects of Ketamine may dissipate more rapidly in young adults when compared to older adults, which may accelerate the required timeline of handoff from Ketamine to SSRI maintenance and introduce a higher risk for dependence [31].

### 2.6. Legality and Recreational Use

Ketamine is a structural derivative of phencyclidine (PCP), synthesized in 1962 and approved by the US Food and Drug Administration in 1970 as a clinical anesthetic. In 2019, the FDA approved the Esketamine nasal spray (Spravato) for Treatment-Resistant Depression (TRD) patients [18]. In 2025, the FDA announced its approval of Spravato as a standalone monotherapy for use with patients with Major Depressive Disorder (MDD). Beyond its role in therapeutic applications, Ketamine has become common in illicit club settings and does retain potential for recreational misuse primarily due to its dissociative effects. Recreational abuse is primarily administered at high doses of >1.0 mg/kg through insufflation. Low doses are reported to induce a sedative, relaxing effect akin to mild traditional anesthetics. High doses may increase the risk of unpleasant overdosing dissociation, in which a person experiences depersonalizing, immobilizing symptoms, and hallucinogenic out-of-body experiences. This risk is not a feasible concern in the clinic, where dose and patient-perceptual states are closely monitored. Prolonged use of illicit Ketamine has been documented to increase the risk of cystitis due to the nephrotoxicity of the compound and its interaction with nephron histology [32]. While also often thought to be compounded by dehydration in party environments, damage to the urinary tract may result in hematuria, hydronephrosis by urethra stenosis, or bladder fibrosis contraction. While this is a well-reported complication of constant, daily illicit use, the proposed therapeutic use of Ketamine within this bridging framework is infrequent, clinically monitored, and uses a higher-purity compound that reduces this risk significantly. Therapeutic dose involves 0.5 mg/kg, usually administered via IV injection, which is a subanesthetic dose that does not risk the higher risks of drug pharmacology found in recreational settings [33].

### 2.7. Psychotherapy Adjunctive Therapy

Ketamine and SSRI co-administration are thought to be accompanied by a window of opportunity for traditional talk-adjunct therapy delivery. The hypothesized mechanism of antidepressant action to increase neuroplasticity may also play a role in facilitating learning, possibly inducing a state of enhanced susceptibility to psychotherapy and cognitive behavioral therapy (CBT). Some early evidence does suggest that tandem talk therapy with Ketamine administration improves response and maintenance goals, in what is now termed Ketamine-Assisted Psychotherapy (KAP) [34]. Within the hospital, licensed therapists (MFTs, LPCs, BCBAs, RBTs) may pair the described bridge protocol with a form of verbal therapy, most commonly cognitive behavioral therapy (CBT) or DBT (dialectical behavior therapy). A common observation of the therapist is that patients often experience a loosening of ego defense called a “psycholytic” status, which allows them to relive and or even overcome otherwise extremely traumatic events that they would normally have difficulty processing [34]. By pairing established professional talk-therapy modalities with the hypothesized neuroplastic window of opportunity, it is hoped that future studies will identify whether this effect is synergistic and create sustained long-term behavioral and mood changes.

### 2.8. Safety Profile

A primary risk cited for Ketamine administration is the possibility of abuse. While safe in controlled settings with a fixed dosage and regulated environment, Ketamine’s risk profile must be responsibly characterized. As a structural analog of phencyclidine (PCP), Ketamine is thought to disinhibit dopamine indirectly through its NMDA receptor antagonism on GABAergic interneurons within the Ventral Tegmental Area (VTA) and the Nucleus Accumbens (NAc), primary sites of dopaminergic regulation [35]. By reducing the inhibitory drive of inhibitory GABAergic interneurons, Ketamine has been associated with a downstream burst of dopamine release in the Nucleus Accumbens. This dopaminergic quick release could induce euphoria and induce abuse through its ties to the traditional mesolimbic reward circuitry pathways. However, a recent study has confirmed that although subanesthetic Ketamine increases reinforcement, dopamine release, and hyperlocomotion in mice studies, it does not significantly alter synaptic plasticity, which is a key feature of addictive drugs [35]. Frequency of dose, magnitude of dose, and administration route will need standardization within future clinical trials and within the controlled therapeutic environment to minimize risk for abuse. Traditional drugs of abuse, such as amphetamines or cocaine, induce a sustained and powerful dopamine level increase within the brain that has been observed to be directly associated with redosing and subsequent addiction [35]. Current evidence suggests that Ketamine’s indirect effect within this short-term bridge protocol in hospitalization settings appears to be insufficient to drive a strong and sustained dopaminergic drive to induce addiction, though this requires further validation [36]. This Ketamine-associated dopamine burst is thought to be regulated in part by autoreceptor feedback via the Dopamine D2 (DRD2) receptors, which detect the surge and inhibit dopaminergic firing. Although this mechanism remains opaque, the risk of Ketamine for therapeutic abuse appears to be relatively low, which may be further reduced by controlled dose and administration within the clinical setting [36]. In multiple RCTs that used sub-anesthetic Ketamine for treating depression, long-term follow-up at around 6 months showed zero cases of novel Ketamine use disorder [37,38].

### 2.9. Ethical Status and Future Directions

Although Ketamine-Assisted Psychotherapy (KAP) and Ketamine + SSRI use have made significant strides in basic science and clinical research settings, there are still several important ethical considerations that have significant implications for the use of the drug. As previously stated, intranasal Esketamine (Spravato) is currently the only formulation that is FDA-approved for psychiatric conditions and must be administered in Risk Evaluation and Mitigation Strategy (REMS)-certified healthcare settings. IV-administration retains the distinct benefit of improved bioavailability by avoiding first-pass metabolism, but it is not yet approved by the FDA for any psychiatric condition. As such, IV Ketamine is frequently used off-label, which exacerbates the previous concerns about patient costs. While generic IV Ketamine is somewhat pharmacologically available, its off-label status excludes it from insurance reimbursement cutouts; this often results in significant out-of-pocket costs that can create a financial barrier to care for many patients [39]. This high cost may exacerbate existing healthcare inequities, in which many low-SES or underserved populations may not have access to a potentially effective therapeutic. Additionally, individuals in this group may suffer from secondary psychosocial stressors and mental health disparities due to the cost of the medication. Future clinical trials might alleviate this issue by performing an efficacy test comparing FDA-approved intranasal Esketamine with off-label IV racemic Ketamine that is frequently used in acute suicidal hospitalization settings. Long-term longitudinal studies that assess cognitive measure trade-offs for maintenance infusions of Ketamine are also needed to establish safety profiles, handoff protocols, and adolescent-specific safety guardrails. This would establish the risk profile of subsequent follow-up IV infusions past the recommended 6-month to 1-year mark and help to determine appropriate handoff timelines to SSRI-mediated relief.

## 3. Discussion

**Figure 4:**
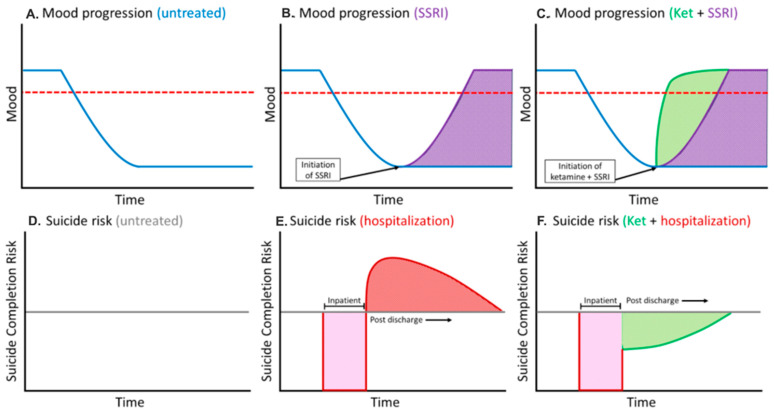
Mood, hospitalization, suicide risk timelines by therapy. (**A**,**D**) represent mood and suicide risk for untreated patients. (**B**,**E**) represent mood and suicide risk after traditional SSRI and psychotherapy SoC. (**C**,**F**) represent mood and suicide risk after SSRI and Ketamine immediate therapy.

Implementing this Ketamine bridge protocol requires a rigorous clinical framework to evaluate therapeutic efficacy and safety. We have created a hypothetical framework of plausible efficacy for Ketamine in filling in the danger zone gaps that current SoC SSRIs appear to create (See Figure 4). The implementation of this strategy for stepdown is hypothesized to mitigate suicide risk in acute psychiatric contexts, though further robust prospective studies are needed to confirm the veracity of this claim. Due to the subanesthetic Ketamine dosage (0.5 mg/kg over 40 min in IV), the care team involved must monitor blood pressure and hemodynamics in real time. Unlike other more traditional sedative agents that usually induce hypotension, Ketamine is a sympathomimetic agent. By indirectly inhibiting the reuptake of stimulatory norepinephrine neurotransmitters, the drug increases heart rate, cardiac output, and blood pressure. Patients’ previous medical history must be fully analyzed for strokes, severe hypertension, or heart failure before administration of Ketamine. Baseline electrocardiogram (ECG) screening should be used to ensure the patient does not have unstable arrhythmias, atrial fibrillation risk (aFib), or severe renal hypertension. Even after the drug has been administered, patient hemodynamics should be monitored by nurses to ensure that Ketamine’s sympathomimetic profile does not induce discomfort or even worsen symptoms due to existing comorbidities. Clinics must maintain consistent electrocardiogram recording and continuous blood pressure monitoring every 20 min to reduce the risk in patients with current or pre-existing cardiovascular complications [36]. Psychomimetic management also remains a concern for this intervention. If a hypertensive crisis occurs due to a combination of existing risk factors with Ketamine’s sympathetic nervous system activation, nursing staff should intervene with other pharmacologic agents, such as beta-blockers, to reduce cardiac output and blood pressure as a safety measure for the patient. Implementing the Ketamine protocol in hospitals requires dedicated resources to train staff, monitor the patient, and safely administer the drug, but the potential for rapid alleviation of severe suicidal behavior may justify these complex operational limitations, if safety protocols are properly adhered to. Patients who are sensitive to dissociative sedatives may be distressed or confused by the experience, particularly if the clinical setting is chaotic. Therefore, the setting should be standardized, predictable, and controlled for maintaining the optimal therapeutic efficacy and ensuring the well-being of the patient. The IV infusion should be in a quiet, darkened room with blackout curtains, calming music, and low-grade warm lighting to minimize the chance of sensory overstimulation [36]. Ideally, care team professionals should also be present to provide verbal prompts to reassure the patient that any auditory or visual hallucinations are temporary. Accounting for all these environmental factors during the proposed hypothetical intervention framework might help to optimize the treatment and assist with its efficacy.

### 3.1. Comparison of Discharge Models

Our proposed Bridge Proposal framework differs conceptually from existing models. Unlike the current Standard of Care (temporary stabilization followed by oral SSRIs) which results in a significant 2–6-week therapeutic latency gap, or Long-Term Ketamine Maintenance (ongoing indefinite infusions with risk of toxicity), the proposed Bridge Protocol utilizes Ketamine by itself as a stop-gap temporary therapy for the 4-week SSRI induction phase until SSRI’s maintenance plasticity is achieved. This minimizes long-term safety risks of long-term Ketamine administration while maximizing safety during the highest-risk window for suicidal behavior.

Despite the progress made on Ketamine as an antidepressant and increased evidence of its mechanistic advantages as an antidepressant, its status as a bridge therapy still remains largely unexplored in the literature and requires rigorous clinical validation. It is still unknown what the optimal dosage or timeline is for transitioning off of Ketamine administration and onto the effects of SSRIs. This “handoff” may be crucial for limiting suicidality and effectively attenuating the suicidal symptomology. Further studies must be performed to determine whether an abrupt termination of Ketamine or a slow reductional taper into SSRI maintenance would be more appropriate for the well-being and mood stability of the patient.

### 3.2. Limitations

It is crucial to acknowledge the limitations of this conceptual review. First, the majority of cited RCTs focus on the acute efficacy of Ketamine rather than the specific post-discharge “bridge” protocol we propose; thus, our framework is an extrapolation of existing data that warrants extensive prospective validation. Second, there is significant heterogeneity across studies regarding dosing (e.g., 0.5 mg/kg vs. fixed doses) and frequency. Third, the transient nature of Ketamine’s effect is a potential weakness; if the pharmacologic handoff to SSRIs is delayed or ineffective, the patient may face a rapid return of suicidality. Strict outpatient monitoring is therefore required to ensure safety during this transition.

## 4. Conclusions

The standard of care for treating acute suicidality in hospital settings currently relies on SSRIs, yet the 2–6-week therapeutic lag creates a critical vulnerability window post-discharge. Current evidence supports the biological plausibility of Ketamine to bridge this gap via rapid mTORC1-mediated neuroplasticity and antidepressant effects. However, the proposed “Bridge Protocol” remains a hypothetical model framework that requires robust clinical validation and presents many pressing procedural questions. Future efforts must focus on conducting pragmatic, longitudinal trials to primarily assess the safety of the handoff between Ketamine and maintenance SSRIs, as well as health–economic analyses to justify the cost. We conclude that Ketamine represents a promising tool that, with careful implementation, might present a new therapeutic option for the management of the post-discharge danger zone for at-risk suicidal patients.

## Data Availability

No new data were created or analyzed in this study. Data sharing is not applicable to this article.
